# Adversity in childhood and later involvement in serious violent crime

**DOI:** 10.23889/ijpds.v8i2.2246

**Published:** 2023-09-14

**Authors:** Alison Teyhan, Rosie Cornish, Kate Tilling, John Macleod, Iain Brennan

**Affiliations:** 1 University of Bristol, Bristol, United Kingdom; 2 NIHR Applied Research Collaboration West (ARC West), Bristol, United Kingdom; 3 University of Hull, Hull, United Kingdom

## Objectives

To use longitudinal birth cohort data linked to police records to examine whether the relationship between adverse childhood experiences (ACEs) and police-recorded serious violence depends on the type, timing or duration of ACEs.

## Methods

The sample are 5070 participants (born 1991-1992) from the Avon Longitudinal Study of Parents and Children who allowed linkage to Avon and Somerset (A&S) local police data, lived in A&S from age 16-24 years, and had exposure and confounder data. The binary outcome (no, yes) is having a police record for a serious violence (SV) offence from age 16-24. ACEs were parent-reported from birth to age 11 and include measures of parental physical and emotional abuse. Logistic regression was used to examine associations between the timing of different ACEs and SV, adjusted for child sex, ethnicity, and family socioeconomic position.

## Results

6% of the participants had experienced physical abuse, 17% emotional abuse, and 121 individuals (2.4%) had at least one SV record. In adjusted models, there was evidence of an association between physical (OR 1.90, 95% 1.08-3.35) but not emotional (0.96, 0.60-1.54) abuse and risk of SV. Results suggest that those who experienced physical abuse in both early (<4 years) and later (4-11 years) childhood, or later childhood only, might have been at greater risk of SV than those who experienced it only during early childhood, although numbers were small and confidence intervals were consequently wide.

## Conclusion

Results to date suggest that associations with SV differ between ACE types, and that timing may be important. In our presentation, we will also present findings for other ACEs.

